# High-performance solid-state ceramic supercapacitors based on novel NASICON-ionic liquid composite electrolyte

**DOI:** 10.1039/d5ra10016j

**Published:** 2026-02-18

**Authors:** Hardeep, Bhargab Sharma, Neha, Kamaldeep Bisht, Anshuman Dalvi

**Affiliations:** a Department of Physics, Birla Institute of Technology and Science, Pilani, Pilani Campus Vidya Vihar Pilani Rajasthan 333031 India adalvi@pilani.bits-pilani.ac.in

## Abstract

A high grain-boundary impedance of sodium superionic conductors (NASICONs) is one of the main obstacles to their use as electrolytes in solid-state energy storage devices, whether batteries or supercapacitors. The present study delves into the use of Na^+^-ion-conducting NZSP (Na_3.45_Zr_2_Si_2_PO_12.225_) in combination with the ionic liquid 1-ethyl-3-methylimidazolium tetrafluoroborate (EMIMBF_4_) in solid-state supercapacitors (SSCs). The optimal composition with ∼12 wt% EMIMBF4 in NZSP exhibits a high ionic conductivity of ∼2.2 × 10^−3^ Ω^−1^ cm^−1^, which is nearly three orders of magnitude higher than that of pristine NZSP. Rietveld refinement confirms the formation of the monoclinic phase of NZSP. *In situ* high-temperature X-ray diffraction further confirms the stability of the composite over a wide temperature range. An optimised electrolyte composition was used to assemble SSCs with ∼1800 m^2^ g^−1^ surface area activated carbon in a lamination cell geometry. The SSC showed outstanding stability, retaining approximately 75% of its capacitance after 15 000 galvanostatic charge–discharge cycles at 2 V and a charge–discharge current density of 1.33 A g^−1^ (2 mA). A typical cell at 2 V/1 mA exhibited a specific capacitance of ∼216 F g^−1^ (50 °C). Further, at 2 V/8 mA discharge current, the symmetric supercapacitor delivers approximately 1970 W kg^−1^ of specific power and about 15 Wh kg^−1^ of specific energy. Supercapacitors exhibited electric double-layer capacitive behaviour at operating potentials ≤2 V. Furthermore, the fabricated devices demonstrated excellent high-temperature operational stability, as evidenced by their reliable operation at 50 °C and 100 °C over 15 thermal cycles. The practical applicability of the fabricated devices is tested by connecting 2 such cells (2 V each) in series, which power a 4 V blue LED for more than 30 minutes.

## Abstract 2

A high grain-boundary impedance of sodium superionic conductors (NASICONs) is one of the main obstacles to their use as electrolytes in solid-state energy storage devices, whether batteries or supercapacitors.

## Graphical abstract

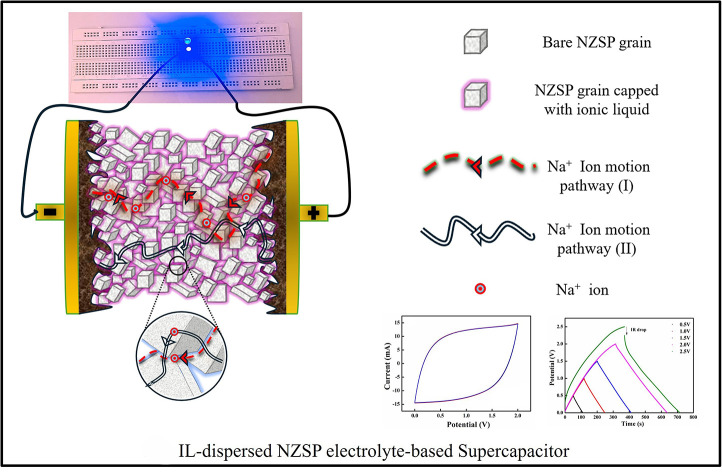

## 1. Introduction

Subsequent to lithium, sodium-ion-based technologies have been considered promising in the field of energy storage, as sodium is the 6th abundant and evenly distributed element in Earth's crust.^1,2^ Despite having similar chemistry to lithium, there are also certain differences, *e.g.* a sodium atom is relatively heavier (23 g mol^−1^) than a Li atom (6.94 g mol^−1^). Further, the redox potential for Na/Na^+^ (−2.71 V *vs.* SHE) is relatively close to Li/Li^+^ (−3.04 V *vs.* SHE) in comparison to other possible electrodes. In addition, a Na^+^-ion is larger (0.98 Å) compared to a Li^+^-ion (0.69 Å).^3,4^ Due to these aforementioned points, the performance of Na^+^-ion batteries is expected not to exceed that of Li^+^-ion batteries in terms of overall performance. This, however, could be less significant provided the primary objective is to achieve a large-scale production at an affordable cost. Na^+^-ion systems are receiving attention after Li^+^-ion systems due to the abundance of sodium, low cost, and global availability. In addition to these advantages, recent advancements in research on Na^+^-ion-based materials have narrowed the performance gap compared to Li^+^-ion systems. At present, research and development efforts are focusing on several promising Na^+^-ion systems. Conventional liquid-electrolyte sodium-ion batteries (SIBs) remain the most widely studied and offer a practical route to commercialisation. At the same time, all-solid-state sodium-ion batteries (ASSIBs) are attracting attention due to their enhanced safety, thermal stability, and long cycle life. Additionally, novel materials, such as newly proposed 2D carbon allotropes for the anode, are being developed to accommodate the large ionic radius of sodium, thereby improving rate capability, structural stability, and overall capacity.^5^ Major challenges researchers face with the current Li^+^ and Na^+^-ion batteries are related to their safety.^6^ Despite advancements, organic liquid-based electrolytes remain in use, which cause leakage and high flammability, and are volatile in nature. Again, designing low-dimensional energy storage systems is not possible using liquid or even gel electrolyte-based technologies. Further, due to limited thermal stability, a wide temperature-tolerant window is also challenging for regular operations.^7–11^ Therefore, devices based on solid-state electrolytes have emerged to fix all these problems. A solid-state electrolyte should have excellent ionic conductivity at room temperature (RT), negligible electronic conductivity, long-term chemical and thermal stability and good compatibility with electrodes.^12,13^ Therefore, it is also a promising candidate for solid-state Na^+^-ion batteries. These solid-state electrolytes (SSEs) include a variety of organic (polymers such as PEO), inorganic (ceramics-based oxide electrolytes) and their composites.^14–17^ Out of these, researchers have given considerable attention to inorganic electrolytes essentially due to their excellent ionic conductivity, high ionic transference number near unity and excellent mechanical and thermal stability.^18^

Among various Na^+^-ion-based SSEs, the most widely investigated inorganic electrolyte was the NASICON-type material. The most explored sodium-based inorganic electrolytes, such as β-alumina, sulphides, hydrides, and NASICONs, exhibit the highest environmental stability. Many of them have been discovered a long time ago. Despite having a good environment stability and a wide electrochemical window, practical applications are not yet realized due to enormously high grain boundary impedance (GBI) and poor interfacial contacts at the electrode–electrolyte (solid–solid) interface.^19^

NASICONs have A_*x*_B_*y*_(PO_4_) general formula, where A is an alkali metal ion, and B is a multivalent metal ion. The Si/PO_4_ tetrahedra share oxygen corners with ZrO_2_ octahedra, forming a 3D interconnected network that accommodates mobile Na^+^- ions. All compounds with this 3D topology are referred to as NASICONs. The NASICON electrolyte with formula Na_1+*x*_Zr_2_Si_*x*_P_3−*x*_O_12_ (0 ≤ *x* ≤ 3) was first proposed by Goodenough and Hong in 1976.^20^ The NASICON compound exhibits either a rhombohedral (*R*-3*c*) phase or a monoclinic (*C*2/*c*) phase depending upon the composition and ambient temperature. At room temperature, Na_1+*x*_ Zr_2_Si_*x*_P_3−*x*_O_12_ (0 ≤ *x* ≤ 3) exhibits a rhombohedral *R*-3*c* space group for all compositions except 1.8 ≤ *x* ≤ 2.2, which shows a monoclinic *C*2/*c* space group. The monoclinic at *x* = 2 exhibits the highest ionic conductivity ∼6.7 × 10^−4^ Ω^−1^ cm^−1^ at RT.^21^ The standard room temperature monoclinic phase of NASICON (Na_3_Zr_2_Si_2_PO_12_) transfers to the rhombohedral phase at ∼160 °C.^22^ There are 6 formula units per unit cell in the rhombohedral phase and 4 in the monoclinic phase. Each formula unit has four inequivalent Na sites, out of which 1 Na_1_ and 3 Na_2_ sites are in the rhombohedral phase, and in the monoclinic phase, there are 1 Na_1_, 1 Na_2_ and 2 Na_3_ sites (3 Na_2_ sites split into 1 Na_2_ and 2 Na_3_).^23,24^ To further improve the ionic conductivity of NASICONs, researchers tried different techniques like doping or substitution of Zr site with different transition metal ions,^25^ adopting advanced sintering techniques such as spark plasma sintering (SPS),^26^ synthesis using different routes such as sol–gel, solid-state reaction (SSR)^27^ and solution-assisted solid-state reaction (SA-SSR).^28^ In addition, adding an excess amount of Na to the standard Na_3_Zr_2_Si_2_PO_12_ (NZSP) NASICON during preparation is an efficient approach that enhances NZSP's conductivity. Despite their high ionic conductivity and good environmental and thermal stability, the potential of Na^+^-ion NASICONs (NZSP) in supercapacitors has been rarely explored to date. Over the past few years, our group, along with others, has attempted to apply some of these fast-ionic ceramics as electrolytes in supercapacitors. A composite of fast-ionic ceramics with polymer electrolytes and ionic liquids has paved the way for their application in supercapacitors. Conductive ceramics, such as garnets and NASICONs, combined with polymer salt complexes, have been successfully applied in supercapacitors. Furthermore, using a minimal amount of ionic liquids (ILs) with ceramics such as garnet, perovskite, and NASICONs, significant improvements in grain boundary conductivity and good interfacial contacts were observed, leading to the development of thermally and mechanically stable supercapacitors.^29^ Ionic liquids are molten salts with a melting point lower than 100 °C and composed entirely of ions that undergo structural variations depending upon the selection of cations and anions. Due to their good thermal stability, low flammability, wide liquid range, low volatility, tunable solubility of both inorganic and organic molecules and wide electrochemical window, they have become very popular in energy storage applications.^30^ Rathore *et al.* conducted a study on the effect of adding the ionic liquid (BMIMBF_4_) to Li^+^-ion oxide glass and their glass ceramics and observed that adding a small amount of ionic liquid (5 wt%) significantly increased the ionic conductivity for a typical glass composition of 60Li_2_SO_4_^−^ 40(0.5Li_2_O–0.5P_2_O_5_) around 2–4 orders of magnitude.^31^ Kaur *et al.* had reported the synthesis of LiTi_2_(PO_4_)_3_ composites dispersed with EMIMBF_4_ IL through the sol–gel method, resulting in conductivity ranging from 10^−3^ to 10^−4^ Ω^−1^cm^−1^.^32^ In a subsequent study, a similar type of NASICON was doped with Al^3+^ to enhance conductivity to ∼10^−3^ Ω^−1^ cm^−1^ and supercapacitors were fabricated using these materials, and approximately 10 000 charge–discharge cycles were performed with nearly 100% efficiency.^33^ Bhargab Sharma *et al.* have recently reported that the Al^+3^-doped LLTO dispersed in a ∼6 wt% EMIMBF4 matrix exhibits 3 orders of magnitude greater capacitance than pristine LLTO, and its supercapacitors with carbon aerogel-based electrodes exhibit excellent cycling stability with ∼87% capacitance retention over 15 000 cycles.^34^

Due to the aforementioned promising properties of ceramic-IL composites, it was deemed important to explore Na^+^-ion NASICON (NZSP) with an IL composite as an electrolyte for supercapacitor applications. Herein, we have developed a Novel NASICON composite by combining Na-excess NZSP with EMIMBF_4_ ionic liquid (IL) in small amount (∼2–12 wt%). The amount of IL in the composite is optimized while examining the NZSP potential in supercapacitors. The Novel composite exhibits a total ionic conductivity of ∼2.2 × 10^−3^ Ω^−1^ cm^−1^ at 50 °C. Various structural and thermal characterization techniques have been used to test the performance of this composite as an electrolyte in solid-state supercapacitor. We evaluated the roles of ILs and Na^+^ ions within the NZSP framework on device performance.

NZSP based ceramic supercapacitors are required to be explored for next generation Na^+^-ion battery-supercapacitor hybrids.

In the present work, our first aim is to present a roadmap in this direction. Thus, to synthesize the Na^+^-ion NASICON with enhanced ionic conductivity, excess Na (∼15wt%) was incorporated to standard Na_3_Zr_2_Si_2_PO_12_ (NZSP) by taking excess amount of precursor having sodium.^27,35,36^ Secondly, to use this Na excess NZSP as an electrolyte in a solid-state supercapacitor (SSC), and for that, a minimal amount (∼2–12wt%) of 1-ethyl-3-methylimidazolium (EMIMBF_4_) ionic liquid (IL) was introduced in Na-excess NZSP powder to improve its grain boundary conductivity and interfacial contact between electrode and electrolyte. Activated carbon (AC) with a high surface area of ∼1800 m^2^ g^−1^ was used as the electrode material. Our work aims to identify and address the factors that affect the performance of these ceramic-IL composite supercapacitors, thereby achieving performance parameters comparable to those of liquid- or gel-based electrolyte supercapacitors.

## 2. Experimental: materials and methods

### 2.1 Materials

Sodium carbonate (Na_2_CO_3_, Molychem 99.9%), trisodium phosphate decahydrate (Na_3_PO_4_⋯12H_2_O, CDH 98%), zirconium dioxide (ZrO_2_, molychem 99.5%), silicon dioxide (SiO_2_, Thermo Scientific 99.995%),1-ethyl-3-methylimidazolium tetrafluoroborate (EMIMBF4, Thermo Scientific, 99%), activated carbon (ASG Scientific) were procured from various distributors and used without further purification.

### 2.2 Preparation: Na-excess NZSP and its composite electrolytes with IL

**Fig. 1:**
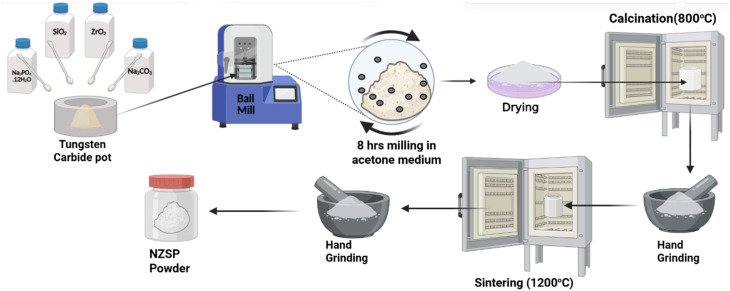
Schematic diagram for the preparation of Na-excess NZSP by solid-state reaction route.

Synthesis of Na-excess NZSP was done by the solid-state reaction (SSR) route. For the preparation, the stoichiometric amounts of Na_3_PO_4_·12H_2_O, ZrO_2_ and SiO_2_ were used. For excess Na content, Na_2_CO_3_ was used, as this only increases the Na content while maintaining a constant stoichiometry for other elements.^27^ The chosen Na content for excess Na was ∼15 wt% because by taking excess ∼15 wt% the standard Na_3_Zr_2_Si_2_PO_12_ (NZSP) system extended to Na_3.45_Zr_2_Si_2_PO_12.225_, which is near the best occupied Na-site of Na_3.4_Zr_2_Si_2.4_P_0.6_O_12_ unit cell (*x* = 2.4).^28,37^ After taking all precursors in stoichiometric ratio, they were mechanically mixed using ball milling in an acetone medium for 6–8 hours. Subsequently, the milled powder was dried at room temperature, and upon complete drying, it was calcined at 800 °C for 12 hours at a heating rate of 5 °C min^−1^ to remove volatile species originating from the decomposition of the sodium carbonate (Na_2_CO_3_) and ammonium dihydrogen phosphate (NH_4_H_2_PO_4_) precursors. Na_2_CO_3_ releases CO_2_ upon decomposition, while NH_4_H_2_PO_4_ thermally decomposes to generate NH_3_ and H_2_O. These gases must be evolved and removed during calcination to avoid residual carbonates, ammonium phosphates, and hydroxides that would otherwise remain in the powder and can disturb the local stoichiometry, impede the solid-state reactions leading to the NASICON structure, and cause porosity and secondary phases that affect the ionic conductivity of the final NZSP ceramics. The calcined sample was pulverised using a mortar and pestle. Again, the grounded white powder was heated to 1200 °C for 12 h, and Na-excess Na_3.45_Zr_2_Si_2_PO_12.22_ was obtained. The schematic representation of the synthesis process is shown in Fig. 1. Na-excess NZSP-IL composites were prepared by uniform mixing of NZSP with EMIMBF_4_ ionic liquid using a planetary ball mill for 1 h in a tungsten carbide pot. The ball-to-sample mass ratio was 5 : 1. The EMIMBF_4_ IL used for composite preparation ranged from 2–12wt%. The samples are abbreviated as NZSP-xIL, where *x* represents the weight per cent (wt%) of IL. The optimal concentration of IL was kept at 12 wt%, because after that, IL started squeezing out from the pellet under high pressure in the die (detailed discussion in Section 2.3).

Experiments with ionic liquid (IL) contents exceeding 12 wt% were avoided, as the goal of this study was to develop an optimised composite solid electrolyte based on sodium-excess NZSP and EMIMBF_4_ for device-related applications. As revealed later (in the inset of Fig. 8(b)), the ionic conductivity saturates at an IL content of 10–12 wt%. Therefore, adding a high amount of IL does not provide any meaningful benefit. Also, the optimised IL concentration in the composite electrolyte for supercapacitor fabrication was 12 wt%. So, most of the measurements, such as XRD, HXRD, FESEM, XPS, and DSC, were performed on the composite containing an optimal ∼12 wt% IL in the Na-excess NZSP-IL composite.

### 2.3 Characterization

X-ray diffraction (XRD) measurements were performed for bare Na-excess NZSP and its IL composite using a Rigaku Miniflex II X-ray diffractometer with Cu Kα radiation (*λ* = 1.54 Å) to identify the phase. The intensity data were recorded between 10° and 90°. FullProf software was used for Rietveld refinement. Structural investigations at elevated temperatures were carried out using *in situ* High-temperature X-ray diffraction (HT-XRD) on a Rigaku Smartlab X-ray diffractometer with Cu Kα radiation (*λ* = 1.54 Å). Field-emission scanning electron microscopy (FESEM) with the FEI Apreo S instrument was used to obtain micrographs for investigating the surface morphology of the composites. Transmission electron microscopy (TEM) using a JEOL JEM-200 operated at 200 kV was used to investigate the internal structure of the composites. Thermogravimetric analysis (TGA) measurements were performed using the DTG-60 series (SHIMADZU) at 10 °C min^−1^, over the 30–800 °C temperature range under a nitrogen atmosphere. X-ray photoelectron spectroscopy (XPS) (Thermo Scientific K-α) was also used to investigate the charge states, oxidation states, and elemental composition of the Na-excess NZSP and Na-excess NZSP-IL composites. The electrical conductivity under steady-state conditions was determined using the HIOKI IM3570 impedance analyzer over a wide frequency range (4 Hz–5 MHz) and a temperature range (50–150 °C). Conductivity calculations were carefully conducted based on the EIS plots generated from the Autolab 204 electrochemical workstation. NOVA Autolab software was used to fit equivalent circuits to EIS data. The composites were pressed at ∼2 tons to form 9 mm-diameter pallets containing *x* wt% IL, abbreviated as NZSP-xIL. After applying conductive graphite paint to the surface, the pellets were kept at approximately 100 °C for 2 hours. Conductive silver paint was applied to both sides of the pellets to ensure electrical contact. Electrical conductivity was then measured using a Hioki Impedance analyser IM-3570 or an Autolab workstation at various temperatures.

### 2.4 Supercapacitor fabrication

**Fig. 2:**
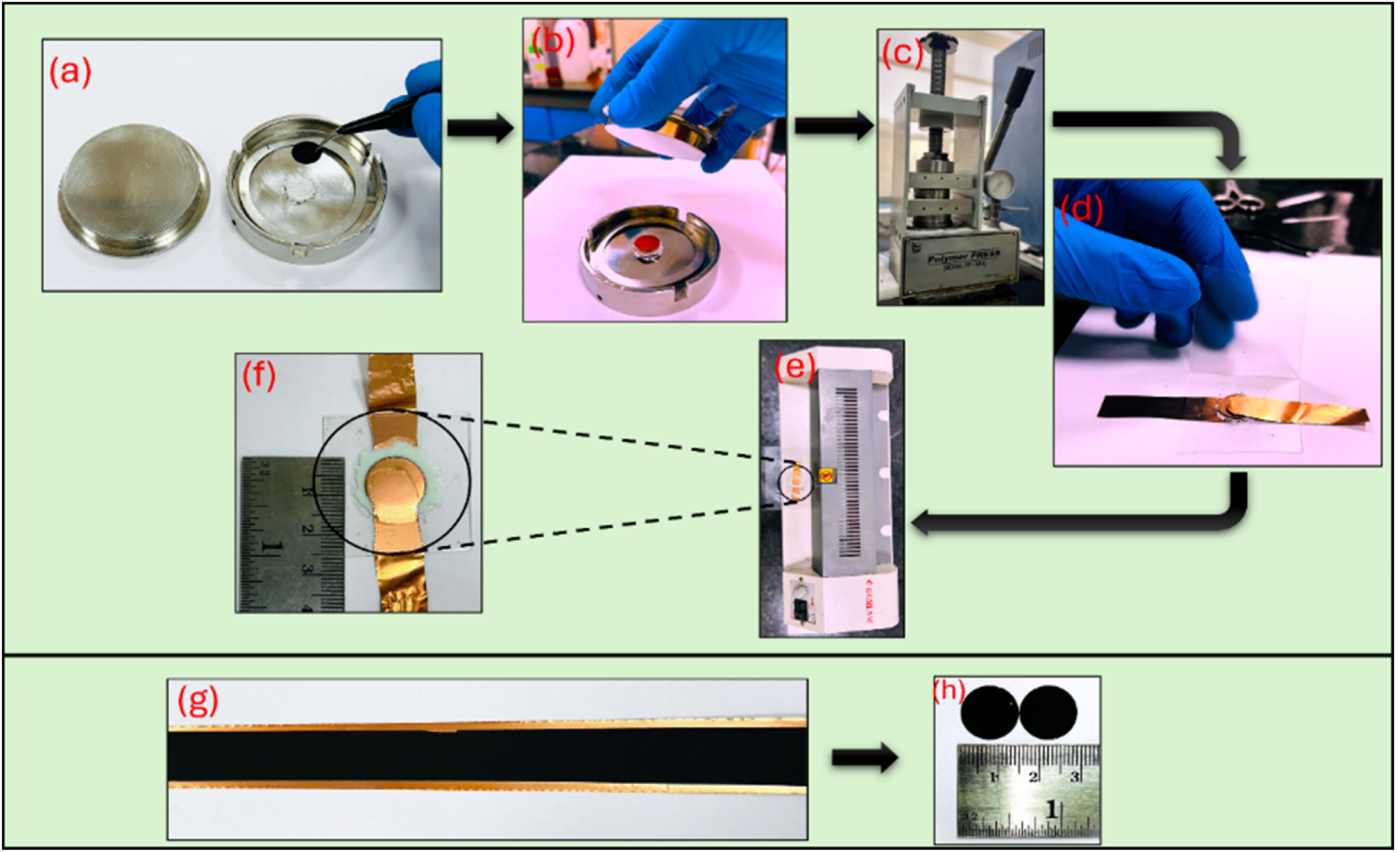
Fabrication steps for SSC using activated carbon (AC) electrodes and Na-excess NZSP-IL composite as electrolyte. (a) Sprinkling composite electrolyte uniformly over AC electrode (b) placing another AC electrode and a spacer to maintain the uniform thickness of electrolyte in between the electrodes (c) pressing the cell with ∼2 tons per cm^2^ in hydraulic press for ∼10 minutes (d) transferring sandwiched geometry between copper connectors and then between the lamination sheet (e) passing through the hot roll lamination machine (f) final laminated supercapacitor for further characterization (g) AC electrode coated on copper collector (h) punched circular shaped electrodes (each of area 1.54 cm^2^) from this coated copper strip.

The Na-excess NZSP-IL composites were further tested under supercapacitor conditions using activated carbon (AC) electrodes (surface area ∼1800 m^2^ g^−1^). The electrode slurry was prepared using AC, PVDF-HFP (binder), and acetylene black (AB) as the electronic conductor in a 80 : 7.5 : 12.5 ratio, respectively. A slurry was coated onto a copper strip (thickness ∼12 microns) using a desktop coating machine (Aero, model AC 250) and subsequently vacuum-dried at 80 °C for about 24 hours. An active mass per electrode was precisely maintained at 1.5 mg, and the electrodes used were punched with a 14 mm puncher (area = 1.54 cm^2^) in SSCs. Na-excess NZSP-12IL composite electrolyte powder was sprinkled uniformly between the AC electrodes and subjected to a pressure of up to ∼2 tons per cm^2^ in a hydraulic press. A spacer was used to maintain an electrolyte thickness of ∼200 µm. The sandwiched configuration was then laminated as shown in Fig. 2 and this assembly was used for further electrochemical characterization. To characterize these SSCs, different techniques such as electrochemical impedance spectroscopy (EIS), cyclic voltammetry (CV), and galvanostatic charge–discharge (GCD) cycling were carried out using an electrochemical workstation Autolab 204. Long Cycling of the SSC was performed using the NEWARE battery testing system.

## 3. Results and discussion

### 3.1 X-ray diffraction

**Fig. 3:**
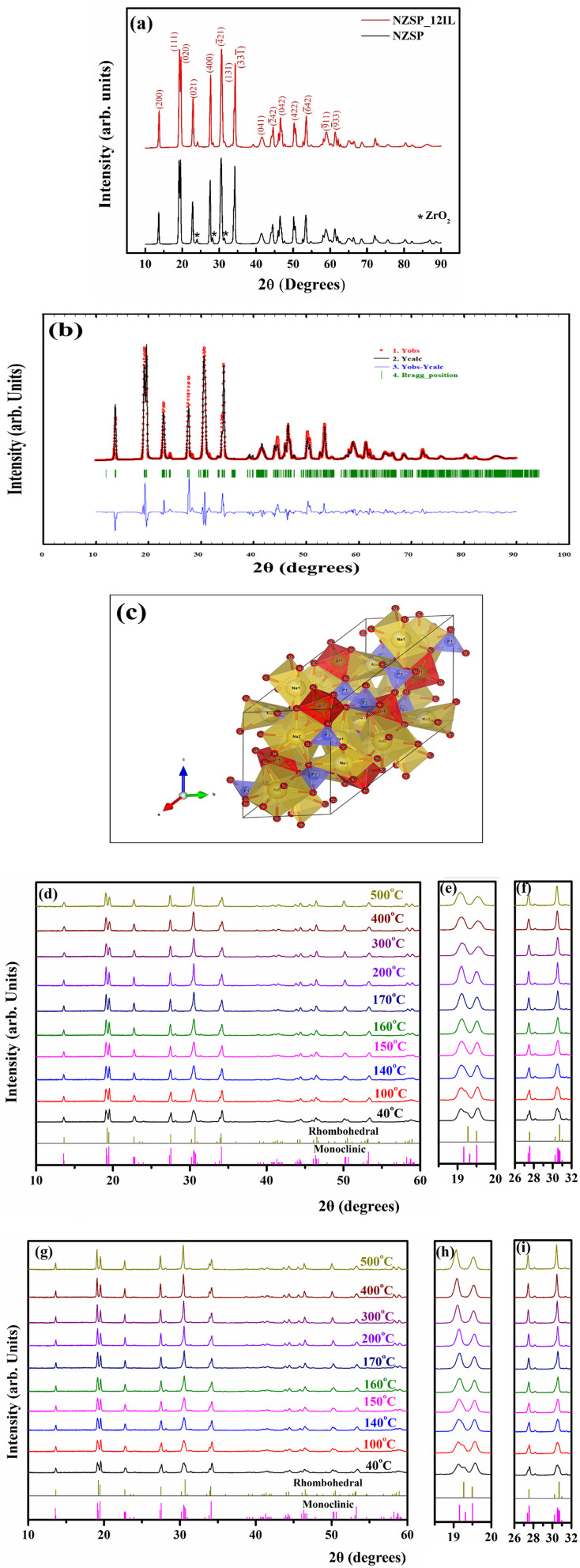
(a) XRD patterns of bare-NZSP and NZSP-12IL composite, (b) Rietveld refinement of Na excess NZSP, (c) crystal structure of Na excess NZSP obtained after refinement, (d) high temperature *in situ* X-ray diffraction (HTXRD) patterns of bare NZSP, (e) and (f) are partial enlargements of (d–g) HTXRD pattern of NZSP-IL composite, (h) and (i) are partial enlargements of (g).

Fig. 3(b) shows the Rietveld refinement of the Na excess NZSP sample at room temperature. The refinement validates the formation of a monoclinic NASICON structure having a *C*2/*c* space group, exhibiting a goodness of fit with a chi-square (*χ*^2^) value of 1.71, demonstrating a good alignment between observed and expected data. Fig. 3(c) represents the structure of monoclinic NZSP, featuring tetrahedra of PO_4_ and SiO_4_ connected to the octahedral corners of ZrO_6_; the structure was obtained using the VESTA software based on the results obtained after Rietveld refinement. The unit cell parameters, which are received after the refinement, are *a* = 15.66 Å, *b* = 9.06 Å, *c* = 9.19 Å, *α* = *γ* = 90°, *β* = 123.89°, and unit cell volume *V* = 1084.58 Å^3^.^38^ These values validate the formation of the monoclinic phase of Na-excess NZSP. Fig. 3(a) demonstrates the XRD patterns for the bare Na-excess NZSP and Na-excess NZSP-IL composite. These apparently exhibit well-defined, sharp peaks, indicating a high crystallinity. Each peak corresponds to a specific set of planes, which are indexed in Fig. 3(a). The average Na-excess NZSP crystallite size, calculated using the Debye–Scherrer relation, is ∼21.6 nm. The ZrO_2_ impurity phase is likely to exist, as inferred from corresponding tiny diffraction peaks at 24.1°, 28.2°, and 31.5°, because it is an inevitable secondary phase and may arise due to the presence of volatile Na and P elements active for reactions during high high-temperature sintering process required for crystal formation or partial reaction of raw materials.^39–41^ The obtained XRD patterns clearly indicate that no new crystalline phase forms when ionic liquid is added to the Na-excess NZSP powder at room temperature. The area under the peak is quite intact, which suggests no loss of the crystalline structure. Additionally, no shift in peaks suggests no change in interplanar spacing (*d*) or lattice parameters, indicating that IL does not enter the NZSP structure and remains at the interface.1
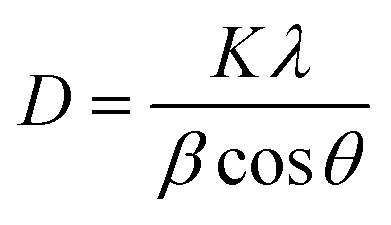
In this context, *D* is the crystallite size (nm), *K* is shape factor, which is usually taken as 0.9 for spherical particles, *λ* is the X-ray wavelength (0.154 nm) for Cu Kα radiation and *θ* (in radians) corresponds to the Bragg angle associated with the diffraction peak and *β* represents the full width at half maximum (FWHM) of the diffraction peak (in radians).

To further investigate the stability of Na-excess NZSP and its composite electrolyte with ionic liquid at high temperatures, *in situ* HTXRD is performed at various temperatures ranging from 40 °C to 500 °C. Fig. 3(d) shows the high temperature X-ray pattern of bare Na-excess NZSP, and Fig. 3(e) and (f) are the partial enlargements of HXRD data of Fig. 3(d) from 18.5° to 20° and 26° to 32°, respectively. As the temperature rises, a gradual merging of the prominent peaks around 19.3°, 27.4°, and 30.5° is observed, confirming the transition from the monoclinic (ICSD:84-1200) phase to the rhombohedral (ICSD:78-1240) phase. The change in structure is due to the rearrangement of Na_2_ and Na_3_ sites in the monoclinic phase to Na_2_ sites in the rhombohedral phase at elevated temperatures. During transition, both phases coexist simultaneously.^22,42,43^ All the diffraction peaks had switched to rhombohedral form above ∼150 °C. Also, from the HXRD pattern of Na-excess NZSP with ionic liquid composite as shown in Fig. 3(g) and its partial enlargement from Fig. 3(h) and (i), we conclude that the NZSP-IL composite electrolyte is relatively stable as a function of temperature, as no new peak is evident with an increase in temperature. At high temperature, IL does not react to form a crystalline compound, and IL also does not react to form an amorphous phase of the NZSP-IL interface, as peaks do not get suppressed. The area under the NZSP peaks remains unchanged, indicating IL physisorption at the interface. Only the phase changes from monoclinic to rhombohedral were observed, as we have previously noticed in bare Na-excess NZSP electrolyte.^22,44^

### 3.2 FESEM analysis

**Fig. 4:**
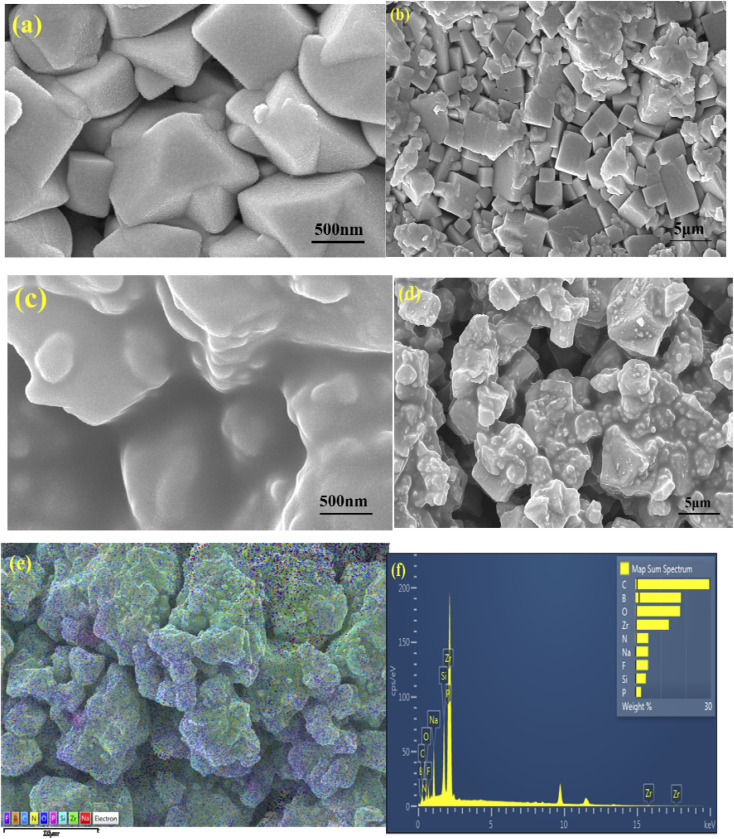
FESEM images of Na-excess NZSP (a) bare at 500 nm magnification, (b) bare at 5 µm magnification, (c) NZSP-12IL at 500 nm magnification, (d) NZSP-12IL at 5 µm magnification, (e) and (f) NZSP-12IL EDS elemental mapping.

To examine the microstructure and morphology of bare Na-excess NZSP sintered powder at 1200 °C for 12 hours, as well as its composition with ionic liquid at room temperature, FESEM images were captured on the powder samples. SEM micrographs at different magnifications are shown in Fig. 4.

Fig. 4(a) and (b) present FESEM micrographs of bare Na-excess NZSP at 500 nm and 5 µm magnification, respectively, revealing the clear growth of NZSP grains without any agglomeration.^44^ In the 5 µm view, one can clearly see very crisp grain interfaces. The Na-excess NZSP with IL composite micrographs are shown in Fig. 4(c) and (d) at 500 nm and 5 µm magnification, respectively. These micrographs suggest that IL simply capped over the NZSP grains without affecting grain size, but the interface is visibly smoother and well-connected. As the grains are not swollen, NZSP-IL composite maintains the structural integrity of Na-excess NZSP by not penetrating the grains but remains localised at grain boundaries. This finding aligns with the XRD results, which show no peak shifts after IL addition.

Energy-dispersive X-ray spectroscopy (EDS) verifies the uniform distribution of all constituent elements in Na excess NZSP and IL. Fig. 4(e) represents the elemental colour mapping of NZSP-IL composite, which clearly shows the chemical homogeneity of Na, Zr, Si, P, O elements from NZSP and F, C, B, and N from the Ionic liquid.

### 3.3 TEM analysis

**Fig. 5:**
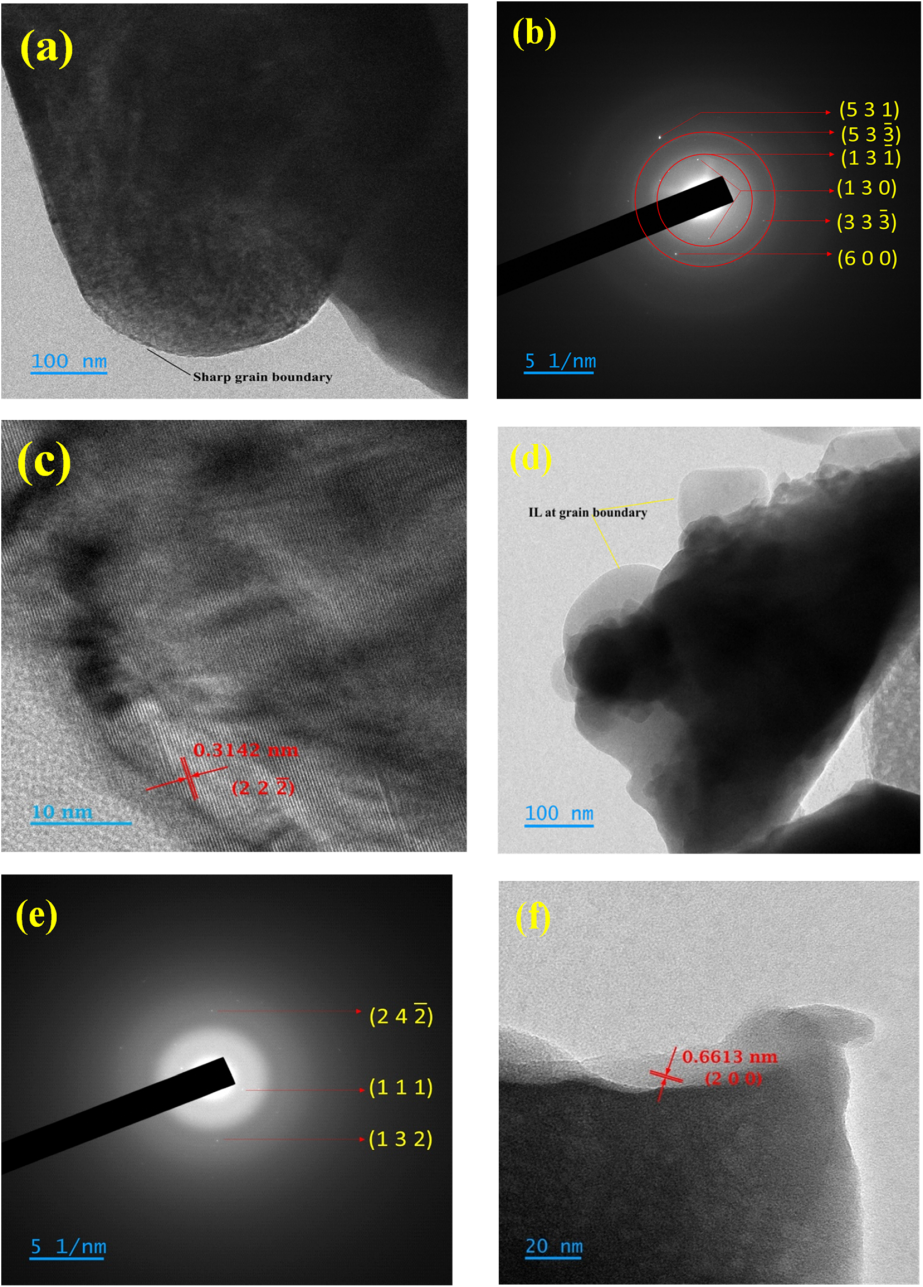
Transmission electron microscopy (TEM) of (a) NZSP (b) SAED of NZSP (c) HRTEM of NZSP (d) TEM of NZSP-IL composite (e) SAED of NZSP-IL composite (f) HRTEM of NZSP-IL composite.

Fig. 5(a) shows the bright field transmission electron microscopy (TEM) image of pristine NZSP, which reveals well-faceted grains with a sharp crystal–vacuum interface and a clear thickness gradient towards the particle edge. The absence of any diffuse rim confirms that the particle surfaces are clean and free from secondary amorphous phases. Fig. 5(b) shows the selected-area electron diffraction (SAED) pattern, which exhibits sharp diffraction spots and rings that can be indexed to higher-order reflections of NZSP, indicating high crystallinity, consistent with the X-ray diffraction (XRD) data. Fig. 5(c) presents the high-resolution transmission electron microscopy (HRTEM) image from the thin edge region, revealing well-defined lattice fringes with an interplanar spacing of 0.3142 nm, indexed to the (222) plane. This confirms the structural integrity and long-range order of the NASICON framework at the nanoscale.

In contrast, Fig. 5(d) presents a TEM image of the NZSP-IL composite, highlighting significant changes in both contrast and particle morphology. The ionic liquid (IL) is uniformly distributed along the grain boundaries, indicating that it has formed a continuous film. Thus, IL at the interface may wet the NASICON grains, effectively eliminating gaps. As a result, the presence of this IL layer in the NZSP likely improves grain boundary contact when compared to the bare sample. In contrast, the sharp grain boundaries of the bare NZSP suggest higher interfacial impedance. Fig. 5(e) displays the selected area electron diffraction (SAED) pattern of the composites, which still shows reflections corresponding to the NZSP planes, confirming that the crystalline phase is preserved. Meanwhile, Fig. 5(f) shows a high-resolution transmission electron microscopy (HRTEM) image of the NZSP-IL composites, in which the lattice contrast appears weakened and spatially interrupted. Notably, a large interplanar spacing of 0.6613 nm, corresponding to the (200) plane, is observed.

Collectively, these observations indicate that while the NZSP framework remains intact after the addition of the IL, the IL forms a conformal amorphous coating that fills the gaps between grains. This filling obscures lattice visibility in HRTEM, broadens the features in the SAED pattern, and alters the TEM contrast. These results provide direct microstructural evidence of a ceramic-IL composite architecture with effective grain boundary infiltration.

### 3.4 Thermal properties: TGA

**Fig. 6:**
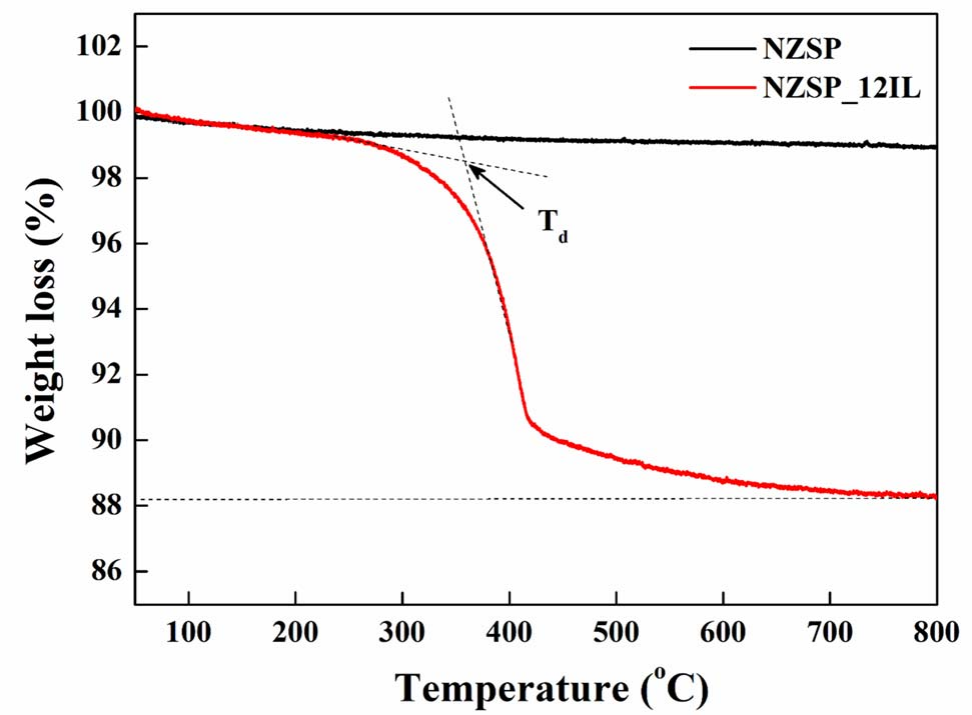
TGA profile of (a) Na-excess NZSP and NZSP-IL composite.

To study the thermal stability and decomposition behaviour of the synthesised composite electrolyte, thermogravimetric analysis (TGA) was performed. Fig. 6 represents the TGA plots for both bare and Na-excess NZSP-IL composite electrolytes, which were recorded from room temperature to 800 °C at a heating rate of 10 °C min^−1^ under a nitrogen atmosphere. A gradual weight loss (∼1%) is observed from room temperature to 800 °C in the bare Na-excess NZSP sample, attributed to moisture evaporation and the sample's phase transition. In case of the NZSP-IL composite electrolyte, minimal weight loss (∼1%) below 150 °C corresponds to the evaporation of adsorbed moisture and volatile impurities, and significant weight loss (∼10%) can be clearly seen in the intermediate region (150–450 °C) in which is attributed to the decomposition of organic entities in the composite electrolyte. The NZSP-IL composite electrolyte gradually loses its weight (∼1.5%) up to 300 °C. As the decomposition temperature (*T*_d_) 360 °C is reached, a significant dip is observed in the curve representing the major weight loss (∼10%) between 300–450 °C.^45^ At temperatures above 450 °C, a gradual weight change (∼1.3%) occurs up to 700 °C, due to the decomposition of carbon residues in the sample. Residual mass above 700 °C indicates the thermally stable oxide phase (*i.e.* NZSP).

In contrast, the TGA curve clearly shows a total weight loss of ∼12% from room temperature to 800 °C, confirming the accurate addition of ionic liquid in the NZSP-IL composite. Also, from the TGA plot, we can say that the NZSP-IL composite is thermally stable up to 250 °C. Sharp dip in NZSP-IL TGA curve between 300–450 °C indicates that ionic liquid is just physically adsorbed on the surface or grain–grain interface of NZSP grains rather than bonded chemically,^34^ which is also corroborated by the XRD and FESEM images. This physisorption allows easy removal of Na^+^ ions, leaving NZSP intact.

### 3.5 XPS analysis

**Fig. 7:**
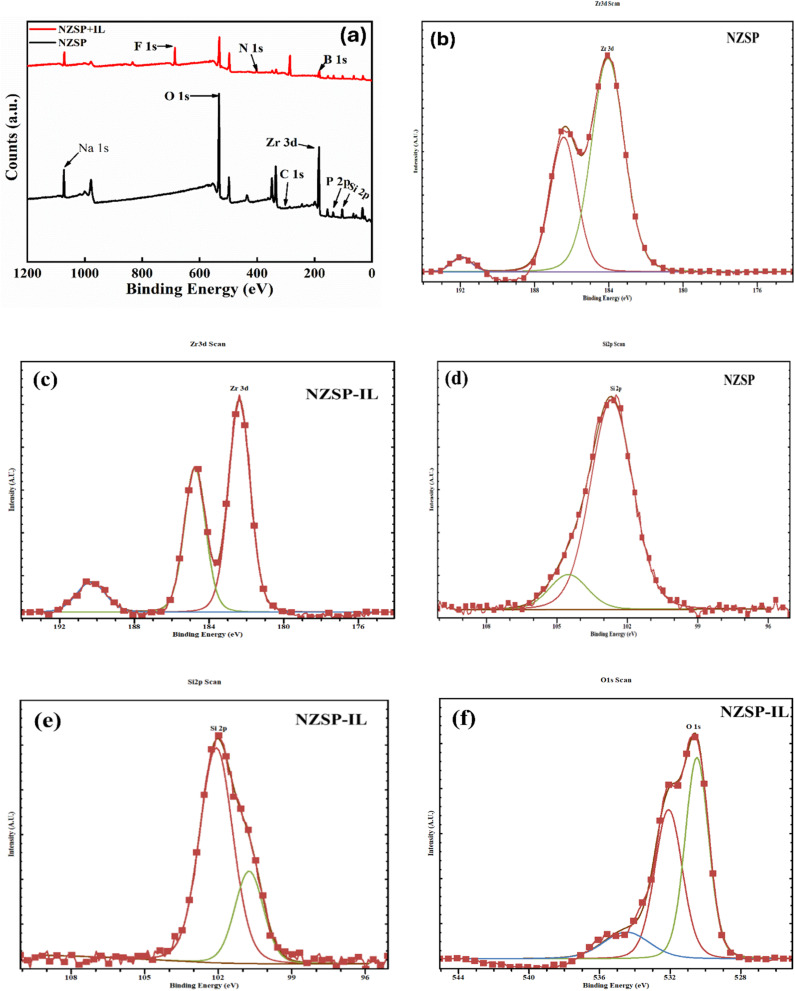
XPS survey spectra of (a) NZSP and NZSP-IL. Deconvolution peaks of (b) Zr 3d for NZSP, (c) Zr 3d for NZSP-IL, (d) Si 2p for NZSP, (e) Si 2p for NZSP-IL, (f) O 1s for NZSP-IL.

X-ray photoelectron spectroscopy (XPS) was used to investigate the surface chemistry and elemental composition of both bare Na-excess NZSP and its composite with EMIMBF4 samples. Fig. 7(a) shows the survey spectrum analysis, conducted across a binding energy range of 0–1200 eV, which revealed characteristic elemental peaks, including Na 1s, Zr 3d, Si 2p, P 2p, O 1s, and C 1s, in both samples.

This confirms the expected elemental composition of the sodium zirconium silicate phosphate framework. Notably, the NZSP-IL sample displayed additional peaks corresponding to F 1s, N 1s, and B 1s, which are characteristic of the ionic liquid presence in composites. The Zr 3d peak in the NZSP-IL spectra is not absent; rather, it is still present. The EMIMBF4 forms a surface layer on the NZSP, which attenuates the photoelectrons emitted from the subsurface Zr. This attenuation follows an exponential relationship as described by the Beer–Lambert law. The presence of these heteroatoms indicates the incorporation of fluorinated anions, nitrogen-containing cationic species (likely imidazolium-based), and boron-containing components. This suggests a complex composition of the ionic liquid that has been effectively deposited onto the surface of NZSP.^46^

A detailed deconvolution analysis of the Zr 3d core-level spectra provided important insights into the oxidation state and chemical environment of zirconium in both samples, as shown in Fig. 7(b) and (c). The Zr 3d spectra displayed the expected spin–orbit splitting, with Zr 3d_5/2_ and Zr 3d_3/2_ components separated by approximately 2.4 eV. This separation is characteristic of the Zr^4+^ oxidation state. In the pristine NZSP sample, the Zr 3d_5/2_ peak appeared at around 182.4 eV, while in the NZSP-IL sample, there was a slight shift to a higher binding energy of approximately 182.6 eV. This subtle shift suggests electronic interactions between the ionic liquid coating and the NZSP surface, potentially through electrostatic interactions or weak coordination bonds with the surface zirconium sites. In pristine NZSP, the Zr 3d region is well described by a single symmetric spin–orbit doublet corresponding to Zr^4+^ in the NASICON lattice. However, in the NZSP-EMIMBF_4_ composite (Fig. 7(c)), the Zr 3d envelope is clearly broadened and asymmetric, showing a distinct high-binding-energy shoulder that cannot be fitted satisfactorily with only one doublet under proper fitting constraints. Therefore, two spin–orbit doublets were used for fitting: the dominant component is assigned to lattice Zr^4+^, while the additional higher-binding-energy component is attributed to surface-modified Zr species such as Zr–OH/ZrO_2_−like environments arising from interfacial interaction and slight surface reconstruction in the presence of the ionic liquid. This indicates surface chemical modification rather than the existence of a second bulk Zr site.^47^

Fig. 7(d) and (e) show a detailed analysis of the Si 2p region, which further confirms the incorporation of silicon into the phosphate framework. The Si 2p spectra exhibited a characteristic doublet structure, with Si 2p_3/2_ located at approximately 102.5 eV and Si 2p_1/2_ at 103.1 eV, showing the typical spin–orbit splitting of about 0.6 eV. The binding energy of Si 2p_3/2_ at 102.5 eV is significantly higher than that of elemental silicon, which is 99.3 eV. This higher binding energy is consistent with the presence of Si^4+^ in an oxidized silicate-phosphate environment. It indicates that silicon is chemically integrated into the NZSP framework rather than existing as separate SiO_2_ phases, thereby supporting the formation of a homogeneous solid solution structure. Additionally, the lack of significant peak shifts between the NZSP and NZSP-IL samples in the Si 2p region suggests that the ionic liquid coating does not significantly affect the bulk electronic structure of the silicon-phosphate network.^48^

Thus, XPS analysis clearly shows that the ionic liquid coating forms a stable interface with the NZSP surface while preserving the structural integrity of the underlying solid electrolyte framework. The binding energy levels for the core elements of NZSP (Zr, Si, P) remain largely consistent between both samples. This indicates that the ionic liquid modification is mainly a surface phenomenon rather than involving bulk intercalation. The surface modification strategy effectively introduces ionic conduction pathways through the organic coating, while maintaining the advantageous electrochemical properties of the NZSP solid electrolyte. The slight electronic changes observed in the Zr 3d spectra suggest favourable interfacial interactions, which may enhance the electrochemical performance of the composite material by improving ionic transport at grain boundaries and electrode interfaces.

### 3.6 Electrical transport

**Fig. 8:**
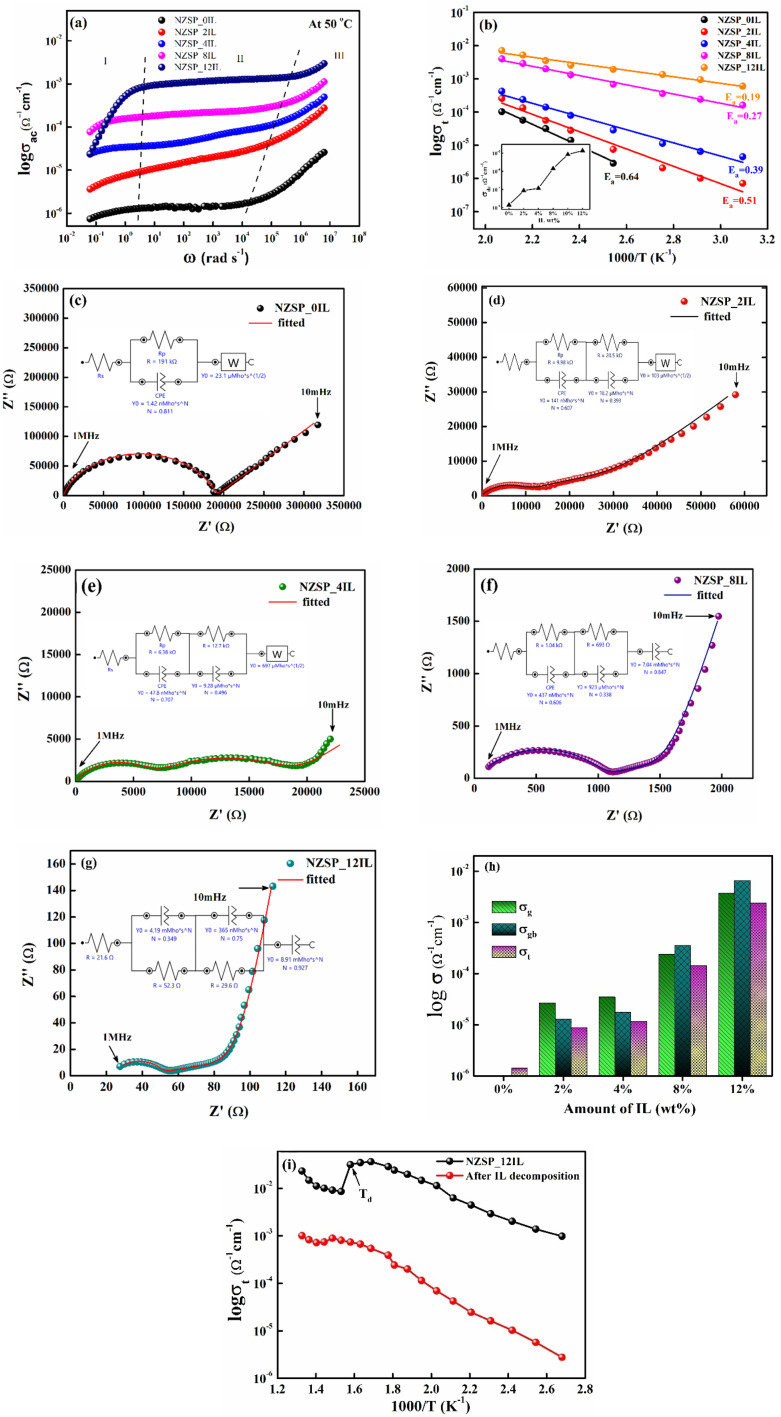
(a) Frequency dependence of electrical conductivity of bare Na-excess NZSP and NZSP_xIL compositions, (b) temperature dependence of total conductivity for all composites with different wt% of IL, Nyquist plot at 50 °C of (c) bare Na-excess NZSP, (d) NZSP_2IL composite, (e) NZSP_4IL composite, (f) NZSP_8IL composite, (g) NZSP_12IL composite, (h) *σ*_g_ (grain conductivity), *σ*_gb_ (grain boundary conductivity) and *σ*_t_ (total conductivity) with different wt% of IL, and, (i) temperature dependence of total conductivity (100 °C–500 °C) for NZSP_12IL sample and the same decomposed sample. The NZSP_0IL correspond to 0 wt% IL sample pellet, which was not sintered before conductivity measurements for a comparative study.

At the outset, the variation in conductivity (*σ*_ac_) *versus* angular frequency (*ω*) at 50 °C is illustrated in Fig. 8(a). Region-I shows a notable fall in conductivity due to ionic polarization at the electrode–electrolyte interface. Furthermore, region II may refer to the DC nature of conductivity, where it exhibits a relatively constant value over a frequency range of almost five orders of magnitude. Region III refers to dc-dispersion, where the conductivity exhibits notable variation with a relatively small change in the *ω* value. This is a typical ionic nature, as witnessed, and from there, the predominant ionic nature can be confirmed. The DC conductivity values are therefore obtained from the plateau.^49,50^ From this plot, it can be clearly seen that pristine Na-excess NZSP powder exhibits low ionic conductivity (∼10^−6^ Ω^−1^ cm^−1^ at 50 °C), and after the incorporation of 12 wt% IL there is a jump of three orders of magnitude (∼10^−3^ Ω^−1^ cm^−1^ at 50 °C) in conductivity. The values of DC conductivity (*σ*_dc_) for the grain and the grain boundary are calculated using the Nyquist plots for different compositions.

The temperature-dependent behaviour of conductivity obtained from the *σ*–*ω* plateau is also studied for different compositions, as shown in Fig. 8(b). In every composition, conductivity increases linearly with temperature, due to a significant reduction in the activation energy (*E*_a_) barrier for ion migration. The bare sample exhibits extremely high grain boundary impedance (GBI), resulting in a very high activation energy (*E*_a_) for ion migration. After capping ceramic grains with an ionic liquid, GBI decreases. Arrhenius behaviour also indicates a single conduction mechanism, without any structural change in the given temperature range. With an increase in wt% of IL from 2 wt% to 12 wt%, a monotonic decrease in *E*_a_ can be observed, as shown in Fig. 8(b). However, after a certain amount (∼12 wt%), IL began to squeeze out of the pellet. The effect of IL addition on conductivity is shown in Fig. 8(b), inset. The trend indicates saturation of conductivity beyond 10 wt% of IL, with no significant increase beyond 10 wt%. However, we used 12 wt% IL during device fabrication to achieve better wettability at the interface and to improve coupling between the electrolyte and the AC electrodes.

The contribution of grain (bulk) and IL at the grain boundary was further associated using impedance spectroscopy (10 MHz–1 MHz). Fig. 8(c–g) shows the complex impedance plots at 50 °C of Na-excess NZSP compositions with 0, 2, 4, 8 & 12 wt% of IL, respectively. The impedance spectra were analysed by fitting the appropriate equivalent circuit using Autolab NOVA software.

In polycrystalline materials, electrical charge transport is governed by three distinct contributions: the grain or bulk contribution, the grain boundary contribution, and the electrode-ceramic interface effect. To elucidate how a polycrystalline ceramic facilitates electrical transport, it was felt important to segregate phase-specific contributions and analyse them by fitting an appropriate equivalent circuit.^51^ In polycrystalline ceramics, such as NASICONs (even perovskites and garnets), grain boundaries are typically more resistive than the grains themselves. As seen from the Nyquist plots (Fig. 8(c–g)), a polarisation tail was observed at lower frequencies, and at mid and high frequencies, depressed semicircles were observed.^52^ While the high-frequency semicircle is associated with bulk or grain conduction, the middle-frequency semicircle is due to grain boundary conduction. The low-frequency tail is due to the longer periodic reversal of the electric field, allowing sufficient time for ionic species to diffuse a greater distance through the electrolyte and polarise at the electrode–electrolyte interface. The equivalent circuit comprising a pure resistor and a pure capacitor in parallel generates a perfect semicircle whose centre lies on the *x*-axis. This type of Nyquist plot is a classical Debye response. The Debye response is the dielectric response of an ideal, non-interacting group of dipoles to an alternating electric field, characterised by a single relaxation time. However, most ionic conductors exhibit a depressed semicircle due to non-Debye-type conduction, indicating a distribution of relaxation times.^53^ To fit Nyquist plots of many real electrolytes, which exhibit depressed semicircles, tilted or curved spikes, a constant phase element (CPE) is used in place of an ideal capacitor in the equivalent circuit,^54^ as shown in Fig. 8 (c–g), where the fitted equivalent circuits are also displayed.

Our focus is to use this Na-excess NZSP NASICON as an electrolyte in supercapacitor fabrication. The Nyquist plot in Fig. 8(a) clearly shows a very high impedance in the bare pellet of the powder sample, which makes it unsuitable for supercapacitor fabrication. The grain boundary impedance is normally large; therefore, it is quite tricky to separate out ingrain and grain boundary contributions.^55^ To tailor the Grain boundary impedance (GBI) of Na-access NZSP, ionic liquid (IL) EMIMBF_4_ was incorporated in NZSP powder. NZSP-xIL composites were formed at different wt% of IL, and their EIS was done as shown in Fig. 8(c)–(g). From Fig. 8(c), it can be seen that the bare sample has a very high (∼191 kΩ) gain boundary impedance. However, with a small amount of IL (∼2 wt%) incorporation, both the grain and grain boundary impedances are dramatically modified. At an optimised (∼12 wt%) weight percentage of IL, the values of *σ*_g_, *σ*_gb_, and *E*_a_ approach an adequate range, allowing us to use NZSP-12IL for electrolytic applications. The evaluated values of *σ*_g_ (grain conductivity), *σ*_gb_ (grain boundary conductivity) and *σ*_t_ (total conductivity) are shown as a bar graph in Fig. 8(h), and their numerical values are also listed in Table 1. It is being observed from Fig. 8(i) that when the temperature dependence of conductivity was measured in the range of 50 °C to 500 °C, there was a continuous increase in conductivity (demonstrating Arrhenius behaviour) up to ∼320 °C. Beyond this point, a sharp decline in ionic conductivity was observed at ∼360 °C. This decrease can be attributed to the decomposition of the ionic liquid, which aligns with the temperature at which a substantial dip was observed in TGA.

**Table 1:** Calculated value of *σ*_g,_*σ*_gb_, *σ*_t_ and *E*_a_ with different wt% of IL at 50 °C

Amount of IL (wt%)	*σ*_g_ (Ω^−1^ cm^−1^)	*σ*_gb_ (Ω^−1^ cm^−1^)	*σ*_t_ (Ω^−1^ cm^−1^)<	*E*_a_ (eV)
Bare	—	—	1.41 × 10^−6^	0.64
2	2.65 × 10^−5^	1.29 × 10^−5^	8.67 × 10^−6^	0.51
4	3.47 × 10^−5^	1.74 × 10^−5^	1.16 × 10^−5^	0.39
8	2.36 × 10^−4^	3.55 × 10^−4^	1.41 × 10^−4^	0.27
12	3.05 × 10^−3^	7.91 × 10^−3^	2.20 × 10^−3^	0.19

The values of *σ*_g,_*σ*_gb_ and *σ*_t_ are calculated as follows:^56^
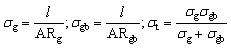


*R*_g_ and *R*_gb_ refer to the bulk and grain-boundary resistances, respectively. Further, *l* and A correspond to pellet thickness and cross-sectional area of the sample pellet.

It may be inferred from the above discussion that:

(1) The incorporation of IL with Na-excess NZSP facilitates the motion of Na^+^ ions across the grains. IL is just capped over the NZSP particles (as seen in SEM images), given that the NZSP bulk structure is stable, as confirmed by XRD and XPS. It is known that ionic liquids have a tendency to dissolve salts due to coulombic interactions between IL ions and salt. Thus, the Na^+^ ions at the interface may get liberated and become mobile. Additionally, the concentration gradient facilitates their motion into and across the interface.

(2) As seen from Table 1, the total ionic conductivity of Na excess NZSP with 12 wt% of IL composite sample shows the highest ionic conductivity ∼2.2 × 10^−3^ (Ω^−1^ cm^−1^) at 50 °C, about three orders of magnitude higher than the bare NZSP at 50 °C. The low ionic conductivity of the pristine sample is most likely due to high interfacial impedance, which hinders Na^+^ ion migration across the grain–grain or electrode–electrolyte interface.^57^ The ionic conductivity of composites is relatively much higher.

(3) In total ionic conductivity of composites, there may be a subtle contribution of ions from the ionic liquid, along with a major contribution from mobile Na^+^ ions liberated from the Na excess NZSP ceramic grains. In many previously reported investigations where the ionic conductivity enhancement by using ILs was achieved, such as Bhargab Sharma *et al.* optimised that by adding ∼6 wt% of EMIMBF_4_ in perovskite type LLTO, the ionic conductivity jumps to the order of ∼10^−3^ Ω^−1^ cm^−1^ at RT.^45^ Further, Kaur *et al.* prepared garnet Li_6.75_Al_0.25_La_3_Zr_2_O_12_ (LALZO) with IL composite, and the optimal composition of ∼3–6 wt% ionic liquid in LALZO showed a high ionic conductivity of 6 × 10^−4^ Ω^−1^ cm^−1^ at room temperature. Li^+^ ion conducting NASICON type LTP and LATP composites with ∼13 wt% of EMIMBF_4_ (IL) show high ionic conductivity of order 10^−3^ Ω^−1^ cm^−1^.^58^ These were used as an electrolyte in 2032-type Li/LiCoO_2_ button-type cells and were stable under battery conditions. These reports suggest that with a minimal amount (∼10–12 wt%) of IL, a significant increase in total conductivity can be seen, but long-range diffusive motion of IL ions is prohibited due to their large ionic sizes. Another investigation into the effect of ionic liquid-dispersed in Li + ion glass and glass ceramics has been conducted by Munesh Rathore *et al.*, finding a significant increase in ionic conductivity with the addition of a very small amount of IL. In a different study on the ionic liquid with Li^+^ ion sulphide glassy system (Li_2_S–P_2_S_5_), it was found that the ionic liquid can dissolve the glass phase when its content exceeds 70 mol%.^59^ Hayashi *et al.* demonstrated the increase in mobility of Li^+^ ion in Li_2_S–P_2_S_5_ glass matrix by the addition of a small amount of EMIMBF4 IL, and the transport number of Li^+^ ion was found to be 0.74, which proved a major contribution of Li^+^ ion to the total ionic conductivity.^60^

(4) To quantify the contribution of ionic liquid in ionic conductivity in Li^+^ ion NASICON LAGP composite with pyr13TFSI (IL) Andrea Paolella *et al.* studied by replacing LAGP by inert Al_2_O_3_ and found three orders of magnitude lower value with respect to LAGP-pyr13TFSI (∼10^−8^ S cm^−1^ at RT).^61^ It gives strong evidence that only ionic liquid ions may not participate effectively in the total ionic conductivity. If so, then pyr13TFSI would have led to a similar value of ionic conductivity as the composition of inert Al_2_O_3._

(5) It was also reported that even after the addition of salt to the ionic liquid, it leads to a decrease in ionic conductivity, rather than an increase. Even if a different ceramic is used with the same IL, a different ionic conductivity (can be high or low) at the same temperature was detected. This demonstrates that ionic conductivity depends upon the compatibility between the ceramic and ionic liquid.^30^ So, one can conclude that Na-excess NZSP and EMIMBF_4_ exhibit superior compatibility, enabling such a high ionic conductivity of order ∼10^−3^ (Ω^−1^ cm^−1^). In fact, the optimised NZSP-12IL composite is used as electrolyte and enables a high-capacity supercapacitor, which is discussed in detail in the next section.

### 3.7 Ceramic supercapacitors using NZSP-IL composites: electrochemical characterization

At the outset, Solid State supercapacitors (SSCs) fabricated using Na-excess NZSP-IL composite electrolytes were characterized using electrochemical impedance spectroscopy (EIS) and Cyclic Voltammetry (CV).

**Fig. 9:**
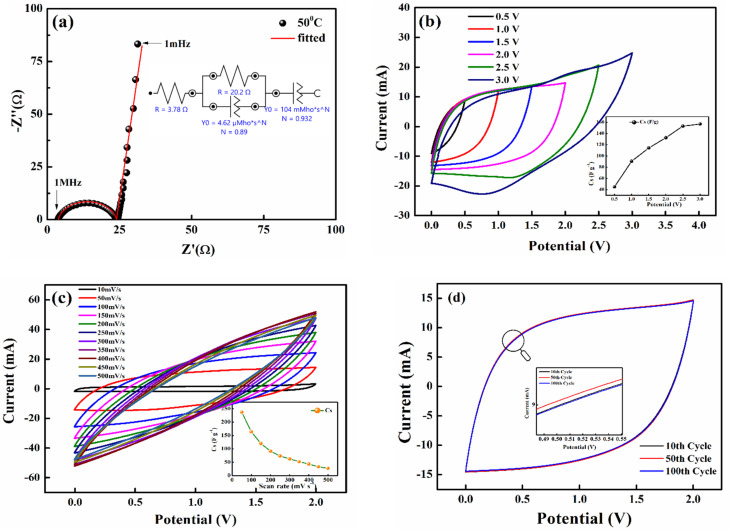
Electrochemical workstation analysis of NZSP-12IL SSC with activated carbon electrodes (a) electrochemical impedance spectroscopy (EIS) of SSC with fitted equivalent circuit; (b) cyclic voltammetry (CV) of SSC at different voltages; (Inset): specific capacitance from CV; (c) CV at different scan rates; (Inset): specific capacitance with different scan rates; (d) 100 CV cycles at 2 V and 100 mV s^−1^ scan rate; (Inset): zoom in view of different cycles.

Fig. 9(a) shows the Nyquist plot for the device over a wide frequency range (1 MHz–1 MHz). The spectrum displays a small value at high frequencies, a depressed semicircle, and a nearly vertical low-frequency tail. The nearly vertical line at low frequencies, having a CPE exponent (*n* = 0.932) close to unity, reflects high capacitive behaviour of the fabricated device.^62^ From equivalent circuit fitting, the series resistance (*R*_s_) or electrode resistance (*R*_e_) of both identical activated carbon electrodes is estimated to be ∼3.78 Ω, and the bulk electrolyte resistance is ∼20.2 Ω. As expected, the electrode resistance is quite low compared with the bulk electrolyte resistance, which contributed most to the internal resistance. The semicircle in the mid-frequency region corresponds to the resistance contribution from the bulk ionic conduction, plus the possible interfacial (charge transfer-resistance).^57,62^ However, the semicircle appears to correspond more closely to bulk electrolyte behaviour, as evident in Section 3.5, where changing the electrolyte conductivity substantially alters the semicircle, indicating that this system is dominated by bulk conduction rather than interfacial charge transfer. Overall, the low resistance values from both electrode and electrolyte indicate well-structured electrolyte–electrode interfaces with reduced internal losses.

Fig. 9(b) shows the cyclic voltammetry (CV) profiles recorded at 100 mV s^−1^ and 50 °C, with the potential window progressively extended up to 3 V to determine the optimal electrochemical stability range. The CV curves remain featureless and nearly rectangular up to 2.5 V, reflecting ideal capacitive behaviour and electrochemical stability. At lower voltages, however, the CV adopts a slightly leaf-like shape, which can be attributed to reduced ionic mobility and sluggish electrode kinetics.^63,64^

The CV curves were also used to assess the capacitance using the formula 
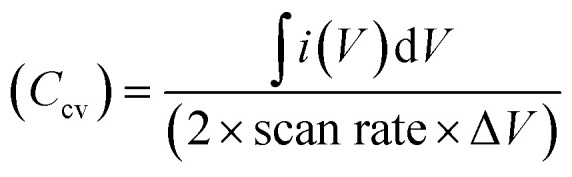
 where ∫*i*(*V*)d*V* is the area under the CV curve, Δ*V* is the potential window (*V*_2_–*V*_1_) used for the scan, and the scan rate is the rate at which the potential (voltage) applied to the working electrode is swept over a specified range The specific capacitance per electrode is then calculated by 
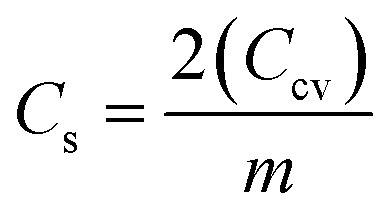
 where, *m* is the average mass of the active material on one electrode. Fig. 9(b) inset presents the specific capacitance (*C*_s_) values derived from the CV curves at different potential windows (Δ*V*). *C*_s_ increase with increase in potential window (Δ*V*) at constant scan rate of 100 mV s^−1^ because area under curve (∫*i*(*V*)d*V*) increase faster than voltage window (Δ*V*). At higher voltages, ions can penetrate deeper into porous electrodes and larger effective area get charged/discharged in electric double layer capacitors. Fig. 9(c) shows CV curves at scan rates ranging from 10 to 500 mV s^−1^ within a 2 V window, demonstrating typical capacitive behaviour even at high scan rates. As the scan rate increases a decrease in *C*_s_ is observed attributed to kinetic limitations. At low scan rate ions have sufficient time to diffuse deep into the electrode pores and access maximum possible active surface area leading to enormous charge storage. However, at high scan rate potential varies very rapidly and ions do not have sufficient time to penetrate deeper in porous electrodes and only outer or easily accessible surface area contribute to charge storage.^64,65^Fig. 9(d) further confirm electrochemical durability, with CV profiles recorded over 100 cycles at 2 V and 100 mV s^−1^ showing negligible degradation, attesting to excellent cycling stability.

**Fig. 10:**
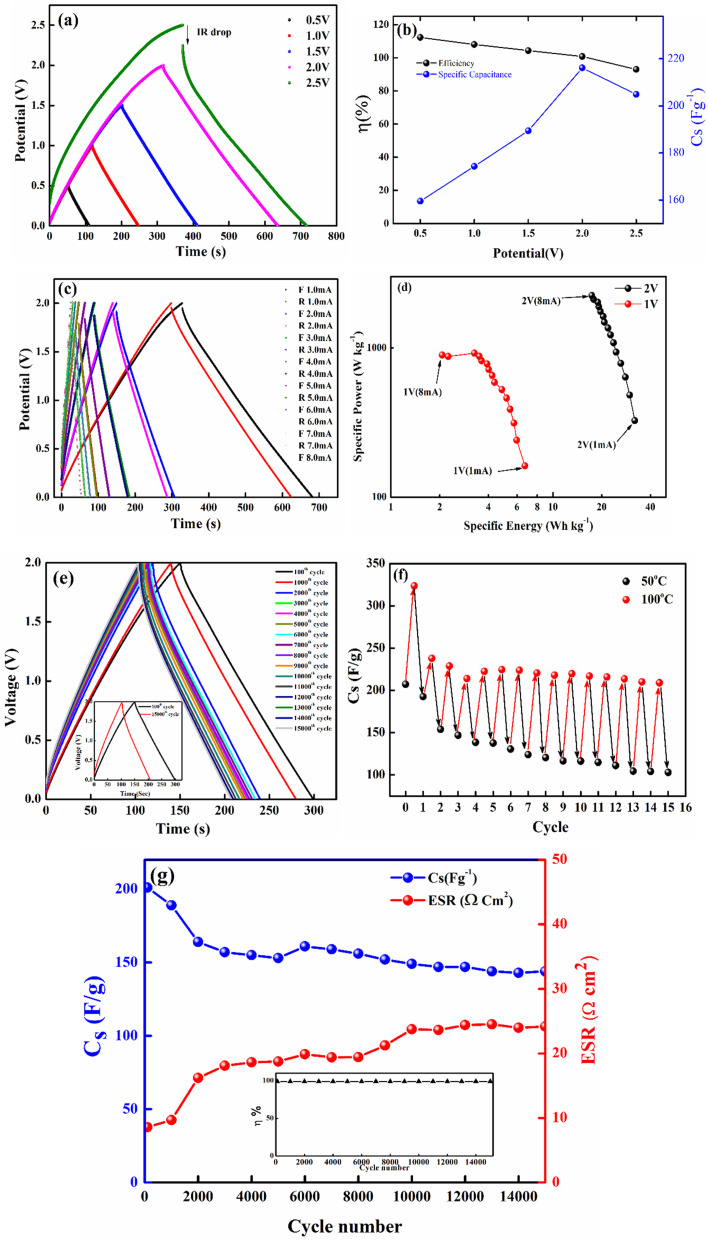
(a) Operating potential optimization of SSC from Galvanostatic charge–discharge (GCD); (b) specific capacitance (*C*_s_) and efficiency (*η*) from GCD at different operating potential; (c) GCD at 2 V with different currents; (d) Ragone plot at 2 V; (e) GCD long cycling up to 15 000 cycles, (Inset): 100th and 15000th cycle; (f) temperature tolerance cycling performance of the device; (g) specific capacitance and ESR (area-normalized) at different cycles, (Inset): coulombic efficiency with cycle number.

The galvanostatic charge–discharge (GCD) cycles were performed at different cut-off voltages to evaluate the operating voltage limit, as shown in Fig. 10(a). The IR drop is quite low for lower potential values. Evidently, at 2.5 V, it reaches 0.255 V. The behaviour of SSC is predominantly EDLC (electric double-layer capacitor) due to the nearly triangular nature of GCD cycles and the box-like nature of CV cycles up to 2 V. Coulombic efficiency 
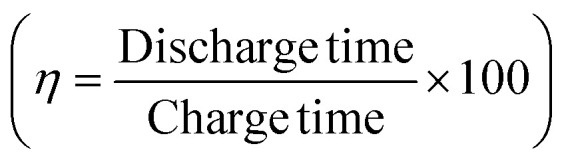
 was obtained from discharging and charging time excluding the IR drop. The galvanostatic charge–discharge (GCD) cycles were also used to evaluate various performance parameters. The total device capacitance 
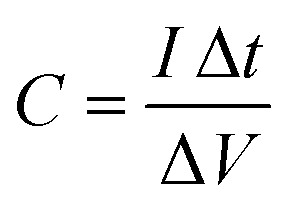
 (in F) is calculated using discharge current (I in A), discharge time (Δ*t* in seconds), and voltage window of the discharge cycle (Δ*V* in volts). The specific capacitance (F g^−1^) per electrode has been calculated as 
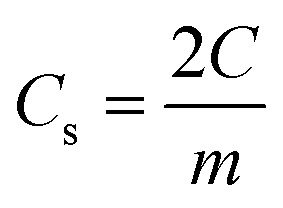
, where *m* is the average mass of active material on a single AC electrode. Moreover, the specific energy 
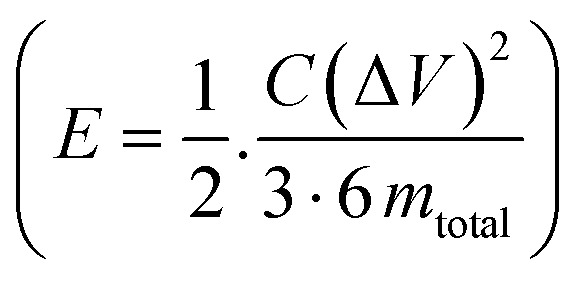
 and specific power 
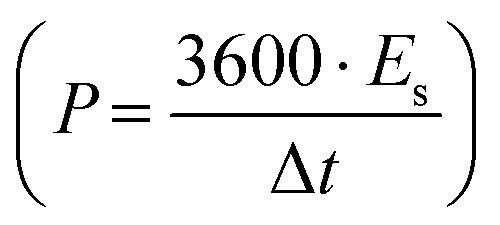
 were obtained for the full device in the units of Wh kg^−1^ and W kg^−1^, respectively. Furthermore, the equivalent series resistance 
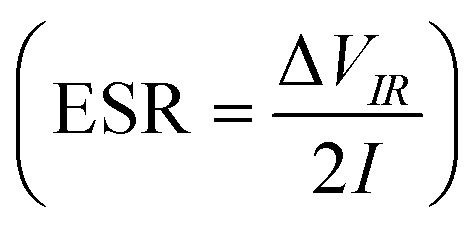
 was calculated using the initial voltage drop during the discharge cycle. The ESR was normalized with respect to the cross-sectional area of the electrode for comparison. Fig. 10(b) illustrates the variation of specific capacitance (*C*_s_) and coulombic efficiency (*η*) with increasing operating potential. At lower voltages, low ionic mobility likely to reduced *C*_s_ values. With increasing potential, *C*_s_ rises monotonically; however, *η* shows a gradual decline up to ∼2.0 V. This drop in coulombic efficiency coincides with deviations from the ideal triangular shape in the galvanostatic charge–discharge (GCD) curves, signalling the onset of non-ideal behaviour or parasitic side reactions. The coulombic efficiency obtained at lower voltages is slightly higher than 100% which is likely due to the inherent nature of AC electrodes. Since AC has different sizes of pores, so during charging at constant current ions do not penetrate into deepest pores immediately and even after charging current stop ions continue their redistribution and migration, and as the discharging starts these delayed ions contribute to additional current, increasing the measured discharge time which results to efficiency >100%.^66^ Based on this, 2.0 V is identified as the optimum working potential for subsequent electrochemical characterization. At this potential, the device delivers a high specific capacitance of ∼216 F g^−1^ (calculated from GCD) at a discharge current of 1 mA, highlighting its strong charge-storage capability under stable operating conditions. Fig. 10(d) presents the Ragone plots for 2 V operation. At 1 mA, the SSC delivers a specific energy of 30 Wh kg^−1^, and at 8 mA, the specific power reaches ∼1970 W kg^−1^.

The SSCs were further evaluated under extreme conditions by recording GCD profiles (20 GCD cycles at each temperature) at 100 °C and 50 °C to assess capacitance retention. Fig. 10(f) presents the cycling (from 50 °C to 100 °C and back to 50 °C, considered as one cycle) of thermal retention performance. Remarkably, even after 15 iterations, the SSC do not degrade and maintains stable operation under these harsh conditions. This demonstrates that the NZSP-IL based SSCs can sustain performance even at elevated temperatures, confirming their robust thermal stability and suitability for high-temperature applications.

The SSC device was operated at an optimized voltage of 2 V/2 mA (1.33 A g^−1^) for long-cycling, since predominant electric double-layer capacitance (EDLC) behaviour is sustained below 2.5 V. Fig. 10(e) shows the galvanostatic charge–discharge (GCD) profiles, which remain stable even after 15 000 cycles. The variation in specific capacitance (*C*_s_) and equivalent series resistance (ESR) with cycle number is shown in Fig. 10(g). The coulombic efficiency *versus* cycle number is plotted in the inset of Fig. 10(g), which shows nearly 100% efficiency throughout the 15 000 cycles. The SSC initially delivers a high *C*_s_ of ∼200 F g^−1^, which gradually decreases to ∼150 F g^−1^ after 15 000 cycles, corresponding to a retention of ∼75%. Throughout the cycling process, coulombic efficiency remains nearly constant at ∼99%, while ESR fluctuates only slightly between 10 and 25 Ω cm^2^. These exciting results confirm excellent electrochemical durability, with a stable electrode–electrolyte interface and negligible degradation over extended operation.

Our group previously conducted thorough investigations into IL composites with LiTi_2_(PO_4_)_3_ (ref. 32)(LTP) and Li_1.3_Al_0.3_Ti_1.7_(PO_4_)_3_ (ref. 67)(LATP). The NZSP-IL composite discussed in this study exhibits high ionic conductivity, comparable to that observed in Li^+^ ion systems. A proposed mechanism for ionic transport in such composites suggests that (i) IL fills the spaces between grains and at the electrode–electrolyte interface, aiding Li^+^ ion movement across grains; (ii) IL ions contribute minimally to overall ionic conductivity due to their larger size and limited mobility when trapped between grains. This explanation aligns with impedance spectroscopy data and initial full-cell tests.^32,67^

Moreover, in this study, the grain boundary impedance of NASICON-IL decreases steadily as the IL content rises, indicating that the IL remains confined between two grains within the structure. The lack of new peaks or shifts in the HTXRD data confirms that the IL does not react with the NASICON matrix. FESEM images support this finding, clearly showing the IL positioned between NASICON grains. Additionally, FTIR analysis (Fig. S1) confirmed this, showing no new bonds formed after adding IL to the NASICON structure.

Most studies indicate that IL primarily involves physisorption on the grain surface, as seen in IL-LTP^32^ or IL-LATP^67^ composites. In the present system, physisorption is quite evident. However, IL can also chemisorb onto the surface, as observed in IL composites with garnet-type Li^+^ ion conductors.^58^ Consequently, the thermal stability depends on the interaction between IL and the grain surface.

**Fig. 11:**
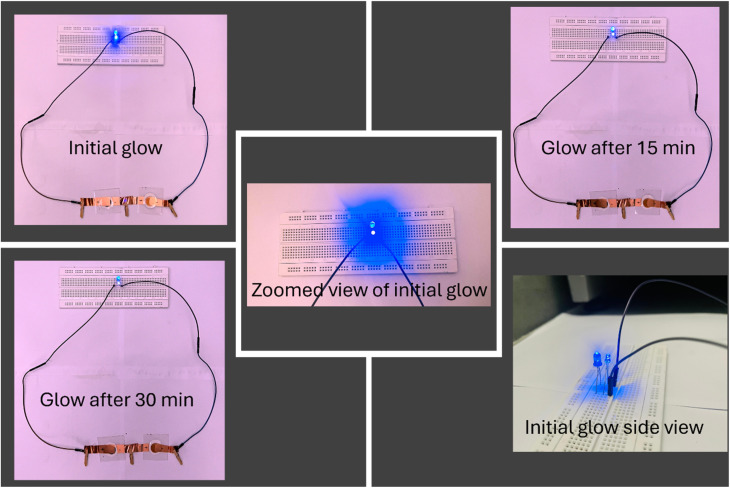
Glow of 4 V blue colour LEDs using two supercapacitors (2 V each) connected in series.

To demonstrate the real-time energy storage capability, the fabricated Na-excess NZSP-IL composite-based devices were employed to power an LED. To power a 4 V blue colour LED, 2 devices have been used which could glow the LED for more than 30 minutes. This highlights their promising potential for real-world energy storage applications (Fig. 11).

## 4. Conclusions

We have demonstrated the first-time development of solid-state supercapacitors using EMIMBF_4_ (IL) modified NASICON-type Na-excess NZSP as an electrolyte. The grain boundary impedance in Na-excess NZSP was successfully tuned by adding a nominal amount of IL, making it suitable for use in solid-state supercapacitors. A high total ionic conductivity of ∼2.2 × 10^−3^ Ω^−1^ cm^−1^ at 50 °C was achieved by adding ∼12 wt% of IL in NZSP matrix. Solid-state supercapacitors were fabricated by sandwiching the novel composite between high-surface-area activated carbon (∼1800 m^2^ g^−1^) electrodes. Structural, morphological and thermal studies of this Novel composite were done by using XRD, FESEM, HXRD, XPS and TGA, and it was found that the structure of NZSP remains unaltered, and IL was just capped over NZSP grains, and no new compound formation was detected, even at high temperature, as corroborated by HXRD and TGA. The present work investigated the potential of the Novel composite as an electrolyte for energy storage in solid-state supercapacitors (SSCs), demonstrating good thermal and mechanical stability. Finally, the optimized solid-state supercapacitor demonstrated remarkable electrochemical stability ∼75% of capacity retention after 15 000 GCD cycles at 2 V and 2 mA (1.33 A g^−1^) with a coulombic efficiency of ∼99%. At 2 V and 8 mA discharge current, the symmetric supercapacitor achieves a specific power of ∼1970 W kg^−1^ and a corresponding specific energy of ∼15 Wh kg^−1^.

The outstanding performance of this novel composite-based solid-state supercapacitor opens the avenue for next-generation energy solid-state storage devices.

## Author contributions

Hardeep: conceptualization, methodology, validation, formal analysis, investigation, XPS, TEM, writing – original draft. Bhargab Sharma: methodology, formal analysis, XPS, TEM. Kamaldeep Bisht: formal analysis. Neha: formal analysis. Anshuman Dalvi: conceptualization, validation, writing – review & editing, funding acquisition.

## Conflicts of interest

The authors declare that they have no known competing financial interests or personal relationships that could have appeared to influence the work reported in this paper.

## Supplementary Material

RA-016-D5RA10016J-s001

## Data Availability

All data included in this work are available upon request by contacting the corresponding authors. Supplementary information (SI): FTIR analysis and Rietveld refinement strategies. See DOI: https://doi.org/10.1039/d5ra10016j.
