# Supplementary material for: High-performance solid-state ceramic supercapacitors based on novel NASICON-ionic liquid composite electrolyte
Source: RSC Adv. 2026 Feb 18;16(11):9500–19. doi: 10.1039/d5ra10016j (PMC12915723; doi:10.1039/d5ra10016j)
Supplement: RA-016-D5RA10016J-s001 [file RA-016-D5RA10016J-s001.pdf]

This journal is © The Royal Society of Chemistry 2026

High-performance solid-state ceramic supercapacitors based on novel NASICON-ionic liquid composite electrolyte

Hardeep, Bhargab Sharma, Neha, Kamaldeep Bisht and Anshuman Dalvi*
Department of Physics, Birla Institute of Technology and Science, Pilani, Pilani Campus, Vidya Vihar, Pilani, Rajasthan 333031, India
*Corresponding author email: adalvi@pilani.bits-pilani.ac.in

FTIR Analysis:

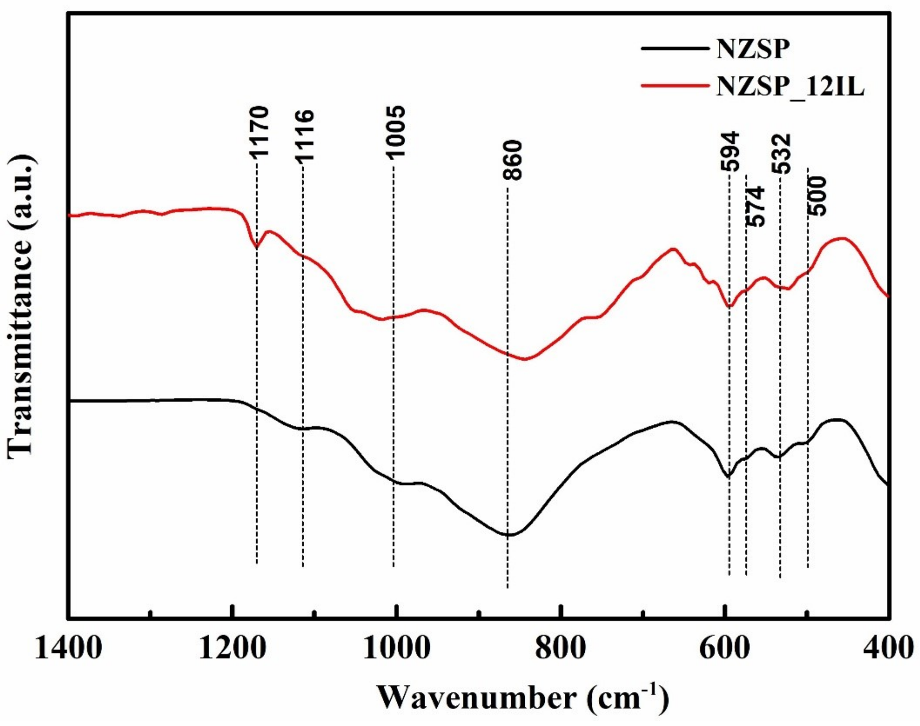

Fig. S1 FTIR of bare-NZSP and NZSP-12IL composite

Fourier transform infrared (FTIR) spectroscopy was used to examine the structural integrity of NZSP and the nature of its interaction with $EMIMBF_4$ in the composite electrolyte. Fig. S1 presents the FTIR spectra of bare-NZSP and the NZSP–$EMIMBF_4$ composite recorded in the range of 400–1400 cm^{-1}. The bands corresponding to 594 cm^{-1}, 574 cm^{-1} and 1115cm^{-1} represent the bending and stretching vibrations of P-O bond in PO_4^{3-} tetrahedral group. While the bands corresponding to 500cm^{-1} and 532cm^{-1} represent the stretching mode of Si-O bond in SiO_4^{4-} tetrahedral groups. These absorption bands confirm the presence of PO_4^{3-} and SiO_4^{4-} tetrahedral

groups in NZSP crystal structure and the presence of these bands confirms the preservation of the NZSP framework, consistent with previous reports on NASICON electrolytes [1] [2]. After incorporation of $EMIMBF_4$, a new prominent band appears at ~1170 cm^{-1} in the composite spectrum, which is absent in bare-NZSP. This band corresponds to the asymmetric B–F stretching vibration of the BF_4^- anion, serving as a fingerprint of BF_4^--based ionic liquids and confirming the successful incorporation of $EMIMBF_4$. Importantly, no new absorption bands or peak splitting is observed, indicating that no new covalent bonds are formed between NZSP and $EMIMBF_4$. These results confirm that $EMIMBF_4$ is physically adsorbed on the NZSP surface, while the NZSP crystal structure remains intact. The composite is therefore stabilised by weak interfacial interactions, which are favourable for enhancing interfacial ion transport without compromising the intrinsic fast-ion-conducting framework of NZSP.

Rietveld refinement strategy

The refinement of the obtained NZSP was performed with FullProf Suite open software with the reference Crystallographic information file obtained from the Crystallography Open Database COD ID 1529608, ref. Baur, W. H.; Dygas, J. R.; Whitmore, D. H.; Faber, J. Neutron powder diffraction study and ionic conductivity of Na~2~Zr~2~SiP~2~O~12~ and Na~3~Zr~2~Si~2~PO~12~ *Solid State Ionics*, 1986, 935-943 and the peaks are fitted with respect to the reference NZSP file.

The refinement was performed with respect to the reference file. Initially, the obtained NZSP spectrum was fitted to the reference data; then the background was fitted, followed by the cell and FWHM parameters; finally, the atom positions and occupancies were refined, yielding a final set of refinement parameters.

The parameters that are refined during the process include scale, cell parameters {lattice constant (a, b, c), angle (α, β, γ)}, full-width half-maxima parameters (U, V, W), background data of the spectrum, atomic positions and occupation. The complete set of refined parameters is provided below.

- Scale factor 0.15257E-04
- Cell parameters

a = 15.66904 b = 9.06594 c = 9.19787

α = 90.00000 β = 123.89285 γ = 90.00000

Vol. = 1084.58 $Å^3$

- Full-width half-maxima FWHM

  U = 0.379993 V = -0.320464 W = 0.099272

- Background polynomial parameter

15.383	0.23774
-6.3223	0.68163
1.6763	1.9980
5.7439	2.0550
-21.237	3.3751
13.566	2.5838

- ATOM PARAMETERS:

Name	x sx	y sy	z sz	B sB	occ. socc.	Mult
Na2	0.50000(0)	0.89100(0)	0.25000(0)	0.000(0)	0.500(0)	4
Na1	0.25000(0)	0.25000(0)	0.50000(0)	0.000(0)	0.405(0)	4
P1	0.00000(0)	0.03920(0)	0.25000(0)	0.000(0)	0.165(0)	4
P2	0.35690(0)	0.11170(0)	0.25860(0)	0.000(0)	0.330(0)	8
Zr1	0.10150(0)	0.24720(0)	0.05390(0)	0.000(0)	1.000(0)	8
Si2	0.35690(0)	0.11170(0)	0.25860(0)	0.000(0)	0.670(0)	8
Na3	0.83600(0)	0.07900(0)	0.84200(0)	0.000(0)	0.600(0)	8
O6	0.08060(0)	0.14750(0)	0.24440(0)	0.000(0)	1.000(0)	8
O5	0.44910(0)	0.18090(0)	0.43690(0)	0.000(0)	1.000(0)	8
O1	0.14660(0)	0.43660(0)	0.22260(0)	0.000(0)	1.000(0)	8
Si1	0.00000(0)	0.03920(0)	0.25000(0)	0.000(0)	0.335(0)	4
O4	0.38120(0)	0.13370(0)	0.11160(0)	0.000(0)	1.000(0)	8
O2	0.43740(0)	0.44390(0)	0.08210(0)	0.000(0)	1.000(0)	8
O3	0.25270(0)	0.18120(0)	0.20280(0)	0.000(0)	1.000(0)	8

After the Rietveld refinement of the NZSP, the obtained values of the fitted parameters are

R-Factors: 14.5 Rp: 19.9 Rwp: 24.3 Rexp: 18.56

Bragg R-factor: 12.82

RF-factor: 6.186

Chi2: 1.72

The experimental values of Rexp and Rwp can be calculated using the following formula[3]

Rexp = $\left[\frac{N-p}{\Sigma_i w_i \left(y_i^{obs}\right)^2}\right]^{\frac{1}{2}} \left\{Where\ w_i = \frac{1}{\sigma_i^2}\right\}$, Rwp= $\left[\frac{\Sigma_i w_i \left(y_i^{obs} - y_i^{cal}\right)^2}{\Sigma_i w_i \left(y_i^{obs}\right)^2}\right]^{\frac{1}{2}}$

N = number of data points

P= number of refined parameters

y_i^{obs} = observed intensity at point i

y_i^{cal} = calculated intensity at point i

$w_i = 1/\sigma_i^2$ = statistical weight

σ_i= standard deviation of intensity

The spectrum was observed at 2°/min scan rate, which may have increased the standard deviation in intensity, resulting in smaller statistical weight and thereby increasing the Rexp value.

The data generated from the refinement are quite similar to the benchmark values for the reported NASICONs, including cell parameters and volume.

a = 15.65130 Å α = 90.0000°

b = 9.05500 Å β =123.7420°

c = 9.21980 Å γ = 90.0000°

V = 1086.5440 Å^3

(ref. Baur, W. H.; Dygas, J. R.; Whitmore, D. H.; Faber, J. Neutron powder diffraction study and ionic conductivity of Na~2~Zr~2~SiP~2~O~12~ and Na~3~Zr~2~Si~2~PO~12~ *Solid State Ionics*, 1986, 935-943 and the peaks are fitted with respect to the reference NZSP file.)

Thus, we can say that despite the relatively high R-factors, which are typical for NASICON materials due to Na disorder and weak scattering, the refined structural parameters (cell constant, atomic positions) are in excellent agreement with literature values.

Apparently, there is a strong correlation between the Sodium occupancy and thermal parameters. As the Sodium ion is disordered in the NASICON, multiple Na sites are vacant. As Sodium is a light and weak X-ray scatterer thus its contribution to the intensity is quite low. This may result in a reduction in diffraction intensity, especially at higher 2theta values. The refinement was performed using isotropic thermal parameters (excluding Biso and Uiso) to converge the structural parameters and avoid over-refinement of the data.

References

1 S. He, Y. Xu, Y. Chen and X. Ma, *J. Mater. Chem. A Mater.*, 2020, **8**, 12594–12602.

2 Y. B. Rao, K. R. Achary, K. K. Bharathi and L. N. Patro, *J. Mater. Sci.*, 2023, **58**, 2222–2233.

3 B. H. Toby, *Powder Diffr.*, 2006, **21**, 67–70.
